# Efficacy and safety of argatroban in patients with acute respiratory distress syndrome and extracorporeal lung support

**DOI:** 10.1186/s13613-017-0302-5

**Published:** 2017-08-03

**Authors:** Mario Menk, Philipp Briem, Björn Weiss, Martina Gassner, David Schwaiberger, Anton Goldmann, Christian Pille, Steffen Weber-Carstens

**Affiliations:** Department of Anesthesiology and Operative Intensive Care Medicine (CCM/CVK), Charité - Universitätsmedizin Berlin, corporate member of Freie Universität Berlin, Humboldt-Universität zu Berlin and Berlin Institute of Health, Campus Virchow-Klinikum, Augustenburger Platz 1, 13353 Berlin, Germany

**Keywords:** ARDS, ECMO, Argatroban

## Abstract

**Background:**

Extracorporeal membrane oxygenation (ECMO) or pumpless extracorporeal lung assist (pECLA) requires effective anticoagulation. Knowledge on the use of argatroban in patients with acute respiratory distress syndrome (ARDS) undergoing ECMO or pECLA is limited. Therefore, this study assessed the feasibility, efficacy and safety of argatroban in critically ill ARDS patients undergoing extracorporeal lung support.

**Methods:**

This retrospective analysis included ARDS patients on extracorporeal lung support who received argatroban between 2007 and 2014 in a single ARDS referral center. As controls, patients who received heparin were matched for age, sex, body mass index and severity of illness scores. Major and minor bleeding complications, thromboembolic events, administered number of erythrocyte concentrates, thrombocytes and fresh-frozen plasmas were assessed. The number of extracorporeal circuit systems and extracorporeal lung support cannulas needed due to clotting was recorded. Also assessed was the efficacy to reach the targeted activated partial thromboplastin time (aPTT) in the first consecutive 14 days of therapy, and the controllability of aPTT values is within a therapeutic range of 50–75 s. Fisher’s exact test, Mann–Whitney *U* tests, Friedman tests and multivariate nonparametric analyses for longitudinal data (MANOVA; Brunner’s analysis) were applied where appropriate.

**Results:**

Of the 535 patients who met the inclusion criteria, 39 receiving argatroban and 39 matched patients receiving heparin (controls) were included. Baseline characteristics were similar between the two groups, including severity of illness and organ failure scores. There were no significant differences in major and minor bleeding complications. Rates of thromboembolic events were generally low and were similar between the two groups, as were the rates of transfusions required and device-associated complications. The controllability of both argatroban and heparin improved over time, with a significantly increasing probability to reach the targeted aPTT corridor over the first days (*p* < 0.001). Over time, there were significantly fewer aPTT values below the targeted aPTT goal in the argatroban group than in the heparin group (*p* < 0.05). Both argatroban and heparin reached therapeutic aPTT values for adequate application of extracorporeal lung support.

**Conclusions:**

Argatroban appears to be a feasible, effective and safe anticoagulant for critically ill ARDS patients undergoing extracorporeal lung support.

## Background

The acute respiratory distress syndrome (ARDS) remains a major burden on intensive care units (ICU) worldwide. Severe cases of ARDS with life-threatening hypoxemia or hypercapnia can undergo extracorporeal membrane oxygenation (ECMO) or pumpless extracorporeal lung assist (pECLA) as a rescue therapy, when conservative treatment strategies such as prone positioning are exhausted and oxygenation cannot be maintained with conventional mechanical ventilation alone. However, this patient population might be particularly challenging with the simultaneous occurrence of heparin-induced thrombocytopenia (HIT). HIT is a serious immune-mediated adverse effect of heparin leading to a pro-thrombotic state [1–3]. Although this occurs in ≤5% of patients receiving heparin, its effects may be fatal, especially in critically ill patients [4]. Mortality rates are high, mostly resulting from thromboembolic events such as venous thromboembolism or myocardial infarction. The clinical diagnosis is based on the presence of thrombocytopenia, typically with a platelet count drop in the first days after heparin administration. Suspicion of HIT must prompt discontinuation of all heparin [5]. Argatroban, a direct thrombin inhibitor, can be used as an alternative anticoagulant to heparin and was shown to improve outcomes in patients with HIT in a prospective, historical controlled study [6].

In ARDS patients a reliable, safe and well controllable anticoagulation strategy is essential to ensure fault-free operation of the lung assist device and to keep complication rates as low as possible. Both thromboembolic events and bleeding are feared complications in ARDS patients undergoing ECMO or pECLA and contribute significantly to mortality [7]. However, knowledge of argatroban in critically ill patients with ARDS is limited. Considering the increasing utilization of lung assist devices for respiratory failure, data on alternative anticoagulation approaches are of growing importance [8, 9].

Given the lack of prospective studies investigating argatroban, the present study aimed to retrospectively evaluate the efficiency, safety and controllability of argatroban compared with standard heparin in ARDS patients treated with extracorporeal lung support.

## Methods

### Setting and patients

This retrospective, observational analysis was conducted at a 14-bed ICU of the department of Anesthesiology and Intensive Care Medicine, Charité-Universitätsmedizin Berlin, a national referral center for treatment of ARDS as part of the German ARDS network (http://www.ardsnetwork.de). After written consent from the data protection officer and the hospital ethics commission (EA1/223/12), all adult patients (≥18 years) admitted for ARDS according to the Berlin definition [10] between January 2007 and December 2014 entered the study. Included were only those patients treated with extracorporeal lung assist devices, i.e., veno-venous extracorporeal membrane oxygenation (VV-ECMO) or pumpless extracorporeal lung assist (pECLA) for more than 2 days. Excluded were patients who underwent VV-ECMO/pECLA for less than 2 days. ARDS treatment followed our local SOPs describing differential indications and duration of advanced therapeutic interventions following specified response criteria as published previously [11]. In short, extracorporeal membrane oxygenation (ECMO) or pumpless extracorporeal lung assist (pECLA) was used as rescue therapy for severely hypoxemic and/or hypercapnic patients, when conservative treatment strategies such as prone positioning or inhalation of nitric oxide (NO) fail to improve the situation. Fast entry criteria for initiation of high-flow ECMO in our treatment algorithm are defined as: (1) paO2/FiO2 < 50 mmHg or (2) SpO2 < 90% for 2 h.

Also identified were all patients receiving argatroban as anticoagulant. As a control group, patients receiving heparin were recruited and were matched with the argatroban group for age, sex, body mass index (BMI) and the severity of illness score SAPS II. After the matching procedure, all the other characteristics of the patients given in Table 1 were compared to show that comparable patient groups were used.

**Table 1:** Basic characteristics of ARDS patients (*n* = 78) on ECMO/pECLA with either argatroban or heparin

	Argatroban (*n* = 39)	Heparin (*n* = 39)	*p* value
Basic characteristics			
Age (years)	47 (36, 60)	48 (35, 64)	0.98
Sex (male) [*n*]	27 (69.2%)	27 (69.2%)	0.99
Body mass index (kg/m^2^)	27 (22.6, 28.7)	26 (23.2, 28.9)	0.80
Severity of illness scores at ICU admission			
SAPS II	56 (40, 71)	58 (40, 76)	0.76
APACHE II	31 (24, 38)	28 (10.7, 38)	0.28
SOFA	13 (9, 14)	12 (9, 15)	0.97
TISS-28	48 (42, 56)	50 (42, 58)	0.53
Severity of ARDS			
Mild (*n*)	9 (23.1%)	4 (10.3%)	0.22
Moderate (*n*)	10 (25.6%)	13 (33.3%)	0.62
Severe (*n*)	20 (51.3%)	22 (56.4%)	0.82
Lung injury score (points)	3.25 (2.75, 3.5)	3.3 (2.8, 3.8)	0.51
Pulmonary gas exchange and mechanical ventilation			
PIP [cm H_2_O]	36 (31, 39)	35 (32, 37)	0.58
PEEP [cm H_2_O]	18 (15, 20)	16 (14, 20)	0.18
Delta P [cm H_2_O]	19 (13, 24)	18 (15, 21)	0.84
FiO_2_	1, 0 (1, 0, 1, 0)	1, 0 (0, 88, 1, 0)	0.23
PaO_2_ [mmHg]	91 (70, 155)	77 (65, 117)	0.26
PaO_2_/FiO_2_	97 (74, 166)	92 (74, 124)	0.40
Pulmonary compliance [ml/cmH_2_O]	25 (15, 35)	23 (17, 36)	0.99
Etiology of ARDS			
Pneumonia (*n*)	18 (46.2%)	19 (48.7%)	0.95
Sepsis (*n*)	1 (2.6%)	4 (10.3%)	0.36
Immune deficiency (*n*)	8 (20.5%)	4 (10.3%)	0.35
Acute on chronic (*n*)	7 (17.9%)	7 (17.9%)	0.99
Trauma (*n*)	4 (10.3%)	0 (0%)	0.12
Others (*n*)	1 (2.6%)	5 (12.8%)	0.20
Extracorporeal lung support			
pECLA (*n*)	9 (23.1%)	15 (38.5%)	0.20
ECMO (*n*)	24 (61.5%)	19 (48.7%)	0.36
ECMO and pECLA (*n*)	6 (15.4%)	5 (12.8%)	0.90
pECLA (h)	164 (117, 258)	220 (116, 393)	0.59
ECMO (h)	265 (131, 460)	428 (180, 652)	0.09
ICU mortality	21 (54%)	22 (56%)	0.34

Discrete variables are presented as median and percentage and were analyzed with Fisher’s exact test for nonparametric samples. Continuous variables are presented as median and 25; 75 percentiles and were analyzed with Mann–Whitney *U* test for nonparametric samples. *APACHE II* Acute Physiology And Chronic Health Evaluation II; *h* hours, *ICU* intensive care unit; *SAPS II* Simplified Acute Physiology Score II. *SOFA* Sequential Organ Failure Assessment, *TISS* Therapeutic Intervention Scoring System. *PIP* positive inspiratory pressure, *PEEP* positive end-expiratory pressure, *delta P* driving pressure, *FiO*_*2*_ fraction of inspired oxygen, *PaO*_*2*_ arterial partial pressure of oxygen, *pECLA* pumpless extracorporeal lung assist, *ECMO* extracorporeal membrane oxygenation; complete data were available for all 78 patients

The observation period for this study was defined as the total period (in days) on ECMO/pECLA and receiving either argatroban or heparin as anticoagulant.

### Management of anticoagulation on extracorporeal lung support and measurements

All included patients received continuous argatroban or unfractionated heparin for anticoagulation while undergoing ECMO or pECLA. Activated partial thromboplastin time (aPTT) was routinely measured three times/day and served as a control variable for anticoagulation. Heparin and argatroban were titrated to an aPTT value of 50–75 s. Argatroban administration was considered upon suspicion of HIT (drop in platelet count, thrombosis), or whenever patients were heparin-non-responsive. Then, heparin infusion was stopped and blood samples were obtained to perform HIT testing. According to our local standard, argatroban infusion was started with an initial dose of 0.3 µg/kg/min, which is much lower than the manufacturer’s recommendation.

### Data collection

Clinical routine data were extracted from the two electronic patient data management systems (PDMS) in use at the hospital (COPRA, Sasbachwalden, Germany; and SAP, Walldorf, Germany).

On ICU admission, in addition to basic demographic data (sex, age, height, weight) and anamnestic data (presence of comorbidities), we assessed ICU admission scores (APACHE II, SAPS II, SOFA, and TISS-28 scores), severity of ARDS according to the Berlin definition, and calculated the lung injury score, as described elsewhere [12]. As major clinical causes leading to ARDS, a differentiation was made between pneumonia, sepsis of extra-pulmonary origin, trauma, immunodeficiency, and ‘acute on chronic’ (i.e., patients with a pre-existing chronic pulmonary disease with acute exacerbation). Also assessed was the utilization and duration of extracorporeal lung assist (ECMO or pECLA).

Data on the following variables were collected for the administration of argatroban or heparin: infusion start time, duration of infusion, and dose used per day. Indications for argatroban (i.e., heparin-induced thrombocytopenia [HIT], heparin non-responder, etc.) were recorded. Clinical outcome and in-hospital mortality were also assessed in order to characterize the patient collective.

The primary safety outcome measures were documented, i.e., significant bleeding and transfusion. Significant bleeding was subdivided into major and minor bleeding complications. Major bleeding was defined as intracranial, pulmonary, retroperitoneal, or gastrointestinal bleeding found clinically or with imaging techniques. Minor bleeding was defined as bleeding at the site of puncture for central venous lines or other catheters, the nasopharyngeal zone, epistaxis, or from skin lesions. We recorded the number of units of packed red blood cells, fresh-frozen plasmas, and platelet concentrates transfused during the observation period, and assessed the amount of blood products (such as fibrinogen and prothrombin complex concentrates) that were administered.

The primary efficacy endpoint was any thromboembolic event associated with the extracorporeal lung assist device, such as clotting of the oxygenator, the pump or of the ECMO/pECLA cannulas with or without necessary replacement. Also recorded was the number of extracorporeal circuit systems replaced during the observation period due to clotting. Secondary efficacy endpoints were new thromboembolic complications (defined as deep venous thrombosis identified by duplex Doppler), pulmonary embolism (diagnosed by computed tomography), limb ischemia, or occlusive stroke found clinically or by imaging techniques.

For evaluation of the controllability and efficiency argatroban and heparin, all targeted activated partial thromboplastin time values (aPTT) were analyzed in the first consecutive 14 days of therapy. We assessed the controllability of aPTT values within the therapeutic range of 50–75 s, and recorded repeated aPTT measurements per day that were necessary to achieve and control for the targeted aPTT corridor.

### Statistical analyses

Discrete variables are given as counts or percentage, and continuous variables as medians with interquartile ranges. For demographics and patient characteristics, differences between the groups were assessed using (retrospective) Fisher’s exact test for categorical variables or Mann–Whitney *U* test for continuous variables when appropriate. Friedman test and multivariate nonparametric analysis for longitudinal data (MANOVA; Brunner’s analysis) were applied for analysis of data over time. Event rates are presented as the mean (±standard deviation) of the (individual) number of events divided by the (individual) time under exposure (days) on ECMO/pECLA multiplied by 100 and were analyzed with the Mann–Whitney *U* test. They reflect the average number of events per 100 patient-days on ECLS in the respective groups. Statistical analyses were performed with IBM SPSS Statistics version 20 (SPSS, Chicago, IL, USA) and SAS version 9.1 (SAS Institute Inc., Cary, NC, USA). Differences were considered significant at *p* < 0.05. All tests should be understood as constituting exploratory data analysis, such that no adjustments for multiple testing have been made.

## Results

### Patient characteristics

**Fig. 1:**
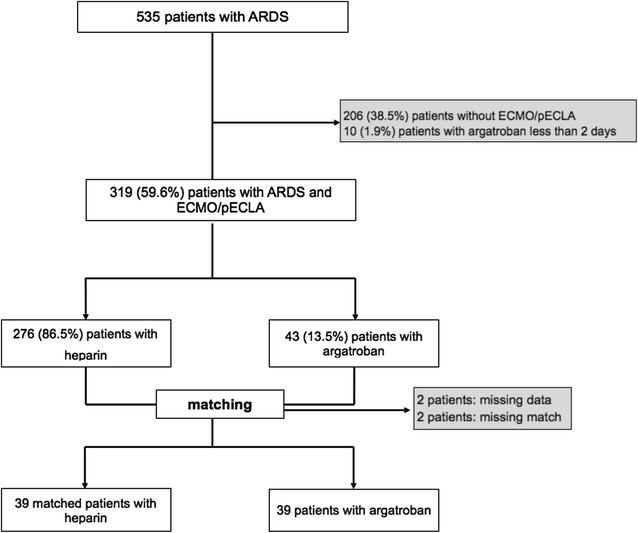
Study population and recruitment of the matched controls

A total of 535 critically ill patients admitted for ARDS were treated between January 2007 and December 2014. Of these, 319 patients (59.6%) underwent extracorporeal lung assist with either ECMO or pECLA. Of this latter group, 43 patients (12.2%) received argatroban for more than 2 days; 4 of these patients dropped out due to missing data or missing matches leaving 39 patients for the analyses. Eventually, we identified 39 matched control patients yielding a total study population of 78 patients (Fig. 1). Characteristics of the study population at baseline are presented in Table 1.

There were no significant differences between the two study groups for age, sex and BMI, or for severity of illness and organ failure scores at ICU admission. Also, applying the three categories of the Berlin definition of ARDS and the lung injury score showed there were no significant differences with respect to severity of lung failure. Parameters of pulmonary gas exchange and mechanical ventilation were similar in both groups. Pneumonia was the most common cause of lung failure. The predominant extracorporeal lung assist procedure was ECMO (55%). Although there was a trend for patients with heparin to be longer on extracorporeal lung support, this did not reach significance.

### Observation period and reason for argatroban

The observation period (i.e., time on extracorporeal lung support with an anticoagulant) was 10 (5; 20) days in the argatroban group and 11 (8; 25) days in the heparin group (*p* = 0.32). Argatroban was started in 13 patients (33%) due to suspected HIT, presenting with typical thrombocytopenia and positive laboratory testing for anti-PF4 antibodies. In total, 26 patients (66%) were non-responsive to heparin, or HIT was suspected but not confirmed. Argatroban was started at a median of 64 (36; 205) h after initiation of extracorporeal lung support.

### Bleeding and thrombotic complications

As primary safety outcome measures, significant major/minor bleeding episodes and thrombotic complications were assessed (Table 2). Overall, major bleeding occurred relatively seldom, with a small number of critical events in both groups. Only 1 patient in the argatroban group and 3 patients in the heparin group developed severe intracranial bleeding while on ECMO or pECLA (*p* = 0.60). In both groups, minor bleedings (e.g., epistaxis or gingival bleeding) occurred significantly more often than major bleedings (47 vs. 217 events in total; *p* < 0.05). However, there were no significant differences in major or minor bleedings between the groups with respect to the number of affected patients or the respective event rates. The incidence of new thromboembolic events (e.g., deep vein thrombosis or pulmonary embolism) was generally low and did not differ between the groups.

**Table 2:** Bleeding, thrombotic complications and replacements of extracorporeal lung support devices of ARDS patients on ECMO/pECLA with either argatroban or heparin

	Argatroban			Heparin			*p* values
	Patients (*n*)	Events. (*n*)	Event rate (mean)	Patients (n)	Events. (n)	Event rate (mean)	*p* (event rates)
Major bleedings: all	11	14	2.7 (±5.3)	13	33	3.8 (±6.8)	*0.57*
Intracranial	1	1	0.07 (±0.4)	3	3	0.7 (±2.6)	*0.24*
Pulmonary	6	8	1.5 (±4.1)	8	17	1.2 (±2.8)	*0.73*
Gastrointestinal	3	3	0.47 (±1.71)	5	8	1.4 (±4.1)	*0.32*
Other	2	2	0.66 (±3.2)	1	5	0.4 (±1.2)	*0.67*
Minor bleedings: all	30	91	19.3 (±19.4)	31	126	22.1 (±23.7)	*0.58*
Catheters. Gingival. Skin	29	84	18.1 (±19.6)	30	116	19.3 (±18.6)	*0.62*
Wounds	2	2	5.0 (±2.4)	9	10	2.8 (±8.2)	*0.06*
Other	1	5	2.8 (17.3)	0	0	0 (±0)	*1.00*
Thrombotic complications: all	6	11	2.5 (±9.9)	3	4	0.8 (±2.5)	*0.54*
Deep vein thrombosis	1	1	0.38 (±2.3)	0	0	0 (±0)	*1.00*
Pulmonary embolism	1	2	0.7 (±4.7)	1	1	0.09 (±0.57)	*1.00*
Cerebrovascular event	1	1	0.3 (±1.6)	1	1	0.2 (±1.3)	*1.00*
Mesenteric infarction	0	0	0 (±0)	0	0	0 (±0)	–
Myocardial infarction	0	0	0 (±0)	0	0	0 (±0)	–
Peripheral artery closure	1	1	0.4 (±2.3)	0	0	0 (±0)	*1.00*
Other	4	6	0.8 (±3.3)	2	2	0.5 (±2.2)	*0.61*
Replacements							
pECLA cannula	0	0	0	0	0	0	–
pECLA oxygenator	8	29	12.9 (±10.1)	10	23	5.4 (±7.5)	*0.17*
ECMO cannula	7	9	1.4 (±3.7)	4	6	2.1 (±7.8)	*0.72*
ECMO oxygenator	15	36	8.5 (±14)	11	21	5.5 (±7)	*0.88*

Discrete variables are presented as absolute numbers of affected patients and absolute numbers of events during the observation period (i.e., the time (days) on ECLS receiving either argatroban or heparin as anticoagulant). Contingency analyses were performed using Fisher’s exact test of retrospective data (no statistically significant differences between the argatroban and heparin group.) Event rates are presented as the mean (±standard deviation) of the (individual) number of events divided by the (individual) time under exposure (days) on ECMO/pECLA multiplied by 100 and were analyzed with the Mann–Whitney *U* test. They reflect the average number of events per 100 patient-days on ECLS in the respective groups

* *p* < 0.05. *Extracorporeal lung support*: *pECLA* pumpless extracorporeal lung assist or *ECMO* extracorporeal membrane oxygenation; complete data were available for all 78 patients

### Extracorporeal lung support device-associated complications

Thromboembolic events associated with the extracorporeal lung assist device (e.g., clotting) occurred infrequently (Table 2). There was no significant difference between the groups with respect to clotting of the inflow/outflow cannulas and with respect to the number of affected patients. Although the number of extracorporeal circuit systems replaced during the observation period due to clotting varied widely, there was no significant difference between the two groups. By trend, replacements of the pECLA oxygenator occurred more frequently in the argatroban group.

### Transfusions, blood products and coagulation factors

There was no significant difference between the groups for units of packed red blood cells, fresh-frozen plasmas and platelet concentrates that were transfused during the observation period on extracorporeal lung support (Table 3). Also, there was no difference in the amount of blood products and coagulation factors that were administered to the two groups. Antithrombin III was significantly more often applied in the heparin group.

**Table 3:** Transfusions and blood products and of ARDS patients on ECMO/pECLA with either argatroban or heparin

	Argatroban (*n* = 39)	Heparin (*n* = 39)	*p* values
	*n* per patient-day on extracorporeal lung support	*n* per patient-day on extracorporeal lung support	
Transfusions			
Packed red blood cells	0.98	1.2	0.61
Fresh-frozen plasma	1.8	1.77	0.86
Platelet concentrate	0.28	0.49	0.21
Blood products and coagulation factors			
Fibrinogen (g)	0.05	0.01	0.60
Prothrombin complex concentrate (IU)	1.96	3.53	0.75
Antithrombin III (IU)	9.7	117	<0.01*

Discrete variables are presented as absolute numbers per patient-day on extracorporeal lung support and were analyzed with Fisher’s exact test for nonparametric samples

* *p* < 0.05; g: gram; *IU* international units; complete data were available for all 78 patients

### Dosing, efficiency and controllability

**Fig. 2:**
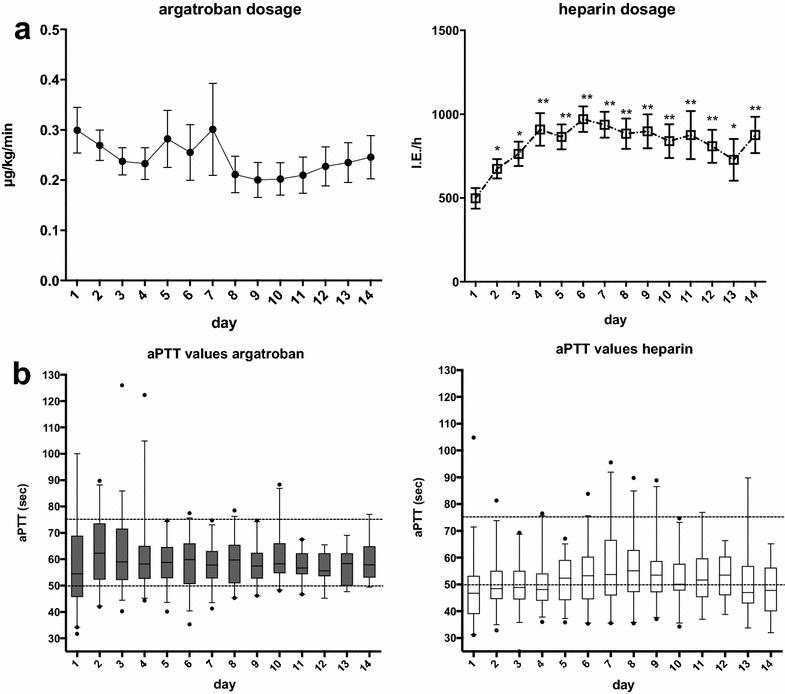
Comparisons of **a** dosage; **b** aPPT values; in ARDS patients undergoing extracorporeal lung support with either argatroban or heparin as anticoagulant in the first consecutive 14 days of therapy *left*: argatroban; *right*: heparin. Data are presented as mean ± standard error of the mean (SEM) and were analyzed with a Mann–Whitney *U* test for nonparametric samples. *p < 0.05; **p < 0.01 compared to day 1. *APTT* activated thromboplastin time; µg/kg/min microgram per kilogram bodyweight per minute; IU/h international units per hour; s seconds; *extracorporeal lung support*: *pECLA* pumpless extracorporeal lung assist or *ECMO* extracorporeal membrane oxygenation

**Fig. 3:**
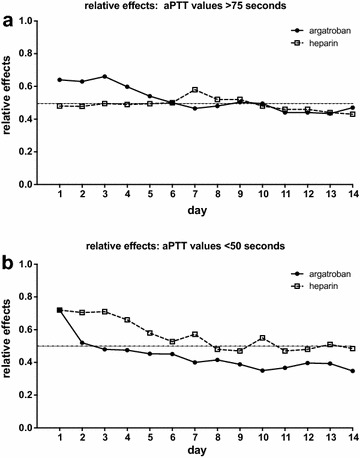
**a** Exceedances of aPTT values >75 s over the course of time in ARDS patients undergoing extracorporeal lung support with either argatroban or heparin as anticoagulant; depicted as relative effect over time; argatroban ****p < 0.0001; heparin *p* = 0.26. **b** aPTT values below 50 s over the course of time, depicted as relative effect over time; argatroban and heparin ****p < 0.0001. The relative effect does not represent the measured values of that parameter, but presents (on a scale of 0–1) the treatment effect of the specific group, relative to all groups and therefore to a ‘mean’ treatment effect. Multivariate nonparametric analysis for longitudinal data (MANOVA; Brunner’s analysis)

The mean starting dose of argatroban was 0.3 (±0.04) µg/kg/min, and the average maintenance dose was 0.26 (±0.038) µg/kg/min. The administered mean dose had to be stepwise decreased in the first three days to maintain an aPTT level below the upper limit of 75 s; (Fig. 2a; left panel). All mean aPTT values were sufficiently within the targeted aPTT corridor (Fig. 2b; left panel). Mean starting dose of heparin was 498 (±336) IU/h, and the average dose was 823 (±124) IU/h. The mean dose of heparin had to be stepwise increased in the first four days to achieve or maintain an appropriate aPTT level; this effect was significant from day 2 onwards (Fig. 2a; right panel). Significantly more aPTT values were below the targeted corridor in the heparin group (heparin 66.5 vs. 33.5% argatroban; *p* < 0.001). Also, mean aPTT values were steadily below the intended 50 s in the first four days of observation (Fig. 2b; right panel). In order to control for aPTT values and to adjust the anticoagulant dose, repetitive measurements of the aPTT were significantly more often made in the argatroban group in the first two days (*p* < 0.05). Repeated aPTT measurements per day decreased significantly over time in both groups; however, this effect was more pronounced in the argatroban group (argatroban *p* < 0.0001; heparin *p* < 0.01). Analyzing the aPTT exceedances over time revealed a significant decrease in the argatroban group, but not in the heparin group (argatroban *p* < 0.0001; heparin 0.26) (Fig. 3a). Also, the number of aPTT values below the lower limit of 50 s showed a significant decrease in both groups over time (argatroban and heparin, *p* < 0.0001) (Fig. 3b).

### Correlation between aPTT and bleeding episodes or thrombotic events

**Fig. 4:**
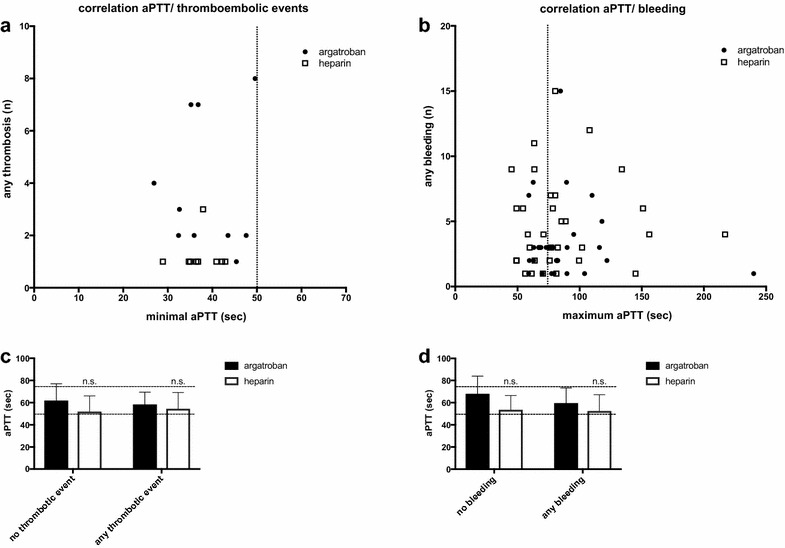
**a** Correlation of any thromboembolic event (*n*) and the minimal aPTT value (*s*) and **b** correlation of any bleeding event (*n*) and the maximal aPTT value (*s*) in ARDS patients undergoing extracorporeal lung support with either argatroban or heparin as anticoagulant (*solid circle* = argatroban; *open square* = heparin). Data are presented as scattered plot; each *dot* represents a patient with anticoagulation and the associated number of thromboembolic episodes or bleeding events, respectively. *Vertical dotted lines* represent the targeted aPTT corridor of 50–75 s. **c**, **d** Comparison of mean aPTT values of ARDS patients undergoing extracorporeal lung support with either argatroban or heparin as anticoagulant with or without thromboembolic events (**c**) and with or without any bleeding episode (**d**). Data are presented as mean ± standard error of the mean (SEM) and were analyzed with a Mann–Whitney *U* test for nonparametric samples. *n.s.* no significant differences between argatroban and heparin group; *n* number; *APTT* activated thromboplastin time; s seconds

The incidence of bleeding did not correlate with the number of aPTT values exceeding 75 s in both groups (argatroban *r* = −0.04, *p* = 0.99; heparin *r* = 0.13, *p* = 0.55). Also, there was no significant correlation between bleeding and the maximum aPTT value (argatroban *r* = −0.10, *p* = 0.54; heparin *r* = 0.03, *p* = 0.84) (Fig. 4b). In both groups, patients with and without bleeding complications showed no significant difference in their mean aPTT values; also, there was no difference between the argatroban and heparin groups (Fig. 4d). Thrombotic events did not correlate with the number of aPTT values below 50 s or with the minimum aPTT value (argatroban *r* = −0.05, *p* = 0.89; heparin *r* = 0.136, *p* = 0.89) (Fig. 4a). Also, mean aPTT values showed no significant difference in patients with and without thrombotic events in both treatment groups (Fig. 4c).

## Discussion

This study focused on the efficacy, safety and controllability of argatroban in patients with ARDS and extracorporeal lung assist devices (ECMO or pECLA). Overall, both anticoagulants enabled therapeutic aPTT values in critically ill patients and were suitable to operate the extracorporeal lung assist device sufficiently and safely, even for a longer period of time. No differences were found between the two groups with respect to bleeding, thrombosis or transfusion. The controllability of both anticoagulants significantly improved over the course of time, with an increasing probability to reach the targeted aPTT corridor.

The present study included patients treated for ARDS in a single center. Matching for standard parameters and severity of illness identified an appropriate control group. ARDS patients in this study were characterized by relatively severe medical conditions, as reflected by high median APACHE II and SAPS II scores at hospital admission. On average, severity scores were higher than in comparable recent studies [13] and even higher than in the ALIVE study [14]. Also, parameters of pulmonary gas exchange and invasiveness of mechanical ventilation indicated severe lung failure. This might be due to our study population, which consisted of very severely affected patients admitted to our ARDS center specifically because their medical condition is poor. On average, more than two-thirds of our patients are transferred from other hospitals [15].

Argatroban was started in 13 patients due to a clinical diagnosis of HIT, presenting with typical thrombocytopenia and positive laboratory testing for anti-pF4 antibodies. This represents about 4% of ARDS patients undergoing extracorporeal lung support, a percentage similar to that reported by others [16]. It is estimated that up to 5% of patients who receive heparin are at risk to develop HIT [4]. However, in the present study, the majority of patients who received argatroban were either non-responsive to heparin or HIT was suspected but not confirmed by laboratory testing. There are many reasons why ARDS patients on extracorporeal lung support may develop a decrease in platelet count; for example, thrombocytopenia might arise due to sepsis, platelet activation or a certain consumption by the extracorporeal circuit. However, the differential diagnosis of HIT in critically ill patients remains a challenge [17] and was not in the scope of our study. Association of a positive 4T score, anti-PF4 and aggregability tests is mandatory to confirm HIT. The threshold to change anticoagulation from standard heparin to argatroban on our ICU is relatively low. Further, patients who were once switched to argatroban upon suspected HIT with ultimately negative HIT testing were not necessarily switched back to heparin. This might explain the relatively high rate of patients receiving argatroban in our study population.

For patients with HIT, the approved initial dose of argatroban is 2 µg/kg/min. Our study population, comprising critically ill ARDS patients with concomitant organ failure, required substantially lower doses of (approximately) 0.26 µg/kg/min argatroban to achieve the therapeutic aPTT goal. This effect might be even more pronounced when the number of failing organ systems increases [18]. Our data are consistent with these findings and also support other studies, which also recommended much lower argatroban doses in critically ill patients [13, 19–21]. There are no specific guidelines for dosing in ARDS patients on extracorporeal gas exchange. In our center, the established starting dose is 0.3 µg/kg/min. The baseline aPTT value, the evaluation of hepatic impairment and the presence of multi-organ-system failure should be taken into account when starting argatroban.

Although major and minor bleeding complications were similar in both groups, there were significantly fewer major bleedings than minor bleedings in both groups. The rates seen in our study are similar to those reported by others [16]. Major bleedings (e.g., pulmonary or intracranial bleedings) were relatively rare events; we found four cases of severe intracranial bleeding, which corresponds to 5% of all our assessed patients. The ELSO reports rates of major intracranial hemorrhage of up to 4% [22]; in most of these cases, the outcome is deleterious. The slightly higher rate found in our study might be explained by the more severe medical condition of our patients, as evidenced by high APACHE and SAPS II scores. Also, impairment of the coagulation system due to sepsis or secondary organ failure, and additional anticoagulation, might render ARDS patients prone to intracranial hemorrhage. However, in this respect, we found no significant differences between argatroban and heparin.

Thromboembolic events are potentially harmful or even life-threatening events in patients with ARDS. Although such events are a major complication of extracorporeal lung support therapy, data on the clinical significance are sparse [23]. As compared to bleeding episodes, thromboembolism rarely occurred in the present study; also, there was no fatality due to thrombotic failure of the extracorporeal lung support device. In the study of Weingart et al., about 25% of patients on extracorporeal lung support needed at least one replacement of the membrane oxygenator [24]. In our study, about 40% of patients needed such replacements. By trend, replacements of the pECLA oxygenator occurred more often in the argatroban group than in the heparin group. We did not observe this with regard to the ECMO oxygenator. However, the overall number of affected patients did not differ significantly between the two groups. Blood flow is much lower in pECLA than in ECMO; also, there may be areas with relative stasis, which can lead to thrombus formation. Nevertheless, the desired ranges of aPTT values in patients with a running system of either kind are comparable for pECLA and ECMO. Therefore, we decided to include both devices in our study.

Interestingly, we found no significant correlation between aPTT values and bleeding or thromboembolic events. Patients with aPTT values exceeding the desired therapeutic range did not necessarily develop bleeding complications. Vice versa, patients with aPTT values below the targeted therapeutic range did not necessarily develop thromboembolic events. However, practically all the thromboembolic events occurred when minimal aPTT values were below 50 s. This is in line with Trudzinski et al., who found an aPTT level below 50 s to be predictive for thromboembolism in ARDS patients under ECMO [25]. On the other hand, almost all bleeding events occurred when the maximal aPTT of the patients was above 50–60 s. Strikingly, more than two-thirds of all bleeding events were associated with maximum aPTT values above 75 s. Although these results only reflect the population of this study, we propose that this aPTT value could be regarded as the utmost tolerable upper limit under extracorporeal lung support to avoid hemorrhage. However, management of anticoagulation in ARDS patients with extracorporeal lung support is not clearly defined. On the basis of our findings, we suggest aPTT levels around 50 s, which is in accordance with most other recommendations and ELSO guidelines suggesting a range 1.5–2.5 times baseline value [19, 21, 24–26]. Development of bleeding might be multifactorial in the intensive care setting and not only dependent on anticoagulation. In particular, impairment of liver function might be held responsible for dysfunction of the coagulation system [27]. Also, volume overload or right-sided heart failure is a typical complication in ARDS patients, which might ultimately lead to hepatic congestion. Overall, in the present study there was no difference between argatroban and heparin with respect to bleeding. Also, there were no significant differences in blood transfusions (a surrogate for blood loss). Our results for ARDS patients on ECMO or pECLA are similar to data from a recent retrospective study based on a prospective cohort database which found an average of one red blood cell per patient-day on VV-ECMO [28]. Thus, argatroban appears to be a safe and suitable anticoagulant in critically ill ARDS patients on extracorporeal lung support.

Finally, we evaluated the controllability of argatroban. Firstly, it enabled therapeutic aPTT values to adequately perform extracorporeal lung support, even for a long time. However, repetitive measurements of the aPTT to adjust for the aPTT goal were significantly more often necessary in the argatroban group in the first four days of therapy. Only a few comparable studies are available on this topic. For example, Treichl et al. [29] report up to three necessary dose adjustments in every patient in order to achieve a distinct aPTT goal. Most other studies do not report details on dose adjustments or aPTT monitoring. Nevertheless, in our study, the controllability of argatroban significantly improved over the first few days, with an increasing probability to reach the targeted aPTT corridor. The mechanism of ‘PTT confounding’ might have been an interfering factor. This condition is defined as a situation where patient-related clinical factors may result in changes in aPTT values that are misleading with respect to indicating the true level of anticoagulation [30]. As a consequence, dosing of argatroban might have been insufficient in some cases.

The present study has some limitations. First, it is retrospective and, although it is one of the largest studies on this topic, the sample size is relatively small. Thus, the study may be underpowered to detect significant differences in mortality, bleeding outcomes or transfusion. Therefore, generalization of these results to other patients undergoing extracorporeal lung support requires considerable caution. Also, in this study the indication for extracorporeal lung support was lung failure. Extracorporeal lung support was primarily cannulated veno-venously, and the results may not allow conclusions to be drawn in the case of a veno-arterial scenario. The patients of this study had very high severity scores. Therefore, there might be different results or thresholds in other patient populations. Another major limitation is that bleeding episodes and thromboembolic events were manually extracted from the patients’ files, which can result in underestimation of the overall incidence. There may be residual confounding that is not captured, such as confounding by indication or indicator. Finally, an additional limitation of the present study is the absence of other parameters of anticoagulation used.

## Conclusion

Argatroban appears to be a feasible, effective and safe anticoagulant for critically ill ARDS patients undergoing extracorporeal lung support. A moderate systemic anticoagulation with aPTT values around 50 s seems to be sufficient to safely operate the lung assist device.

## List of abbreviations

ARDS: Acute respiratory distress syndrome
aPTT: Activated partial thromboplastin time
MANOVA: Multivariate nonparametric analyses of longitudinal data
VV-ECMO: Veno-venous extracorporeal membrane oxygenation
HIT: Heparin-induced thrombocytopenia
pECLA: Pumpless extracorporeal lung assist
ICU: Intensive care unit
BMI: Body mass index
SAPS: Simplified Acute Physiology Score
APACHE: Acute Physiology And Chronic Health Evaluation
SOFA: Sequential Organ Failure Assessment
TISS-28: Therapeutic Intervention Scoring System-28
PIP: Positive inspiratory pressure
PEEP: Positive end-expiratory pressure
FiO_2_: Fraction of inspired oxygen

## References

[1] Nand S, Wong W, Yuen B, Yetter A, Schmulbach E, Gross Fisher S (1997). Heparin-induced thrombocytopenia with thrombosis: incidence, analysis of risk factors, and clinical outcomes in 108 consecutive patients treated at a single institution. Am J Hematol.

[2] Visentin GP, Moghaddam M, Beery SE, McFarland JG, Aster RH (2001). Heparin is not required for detection of antibodies associated with heparin-induced thrombocytopenia/thrombosis. J Lab Clin Med.

[3] Greinacher A, Ittermann T, Bagemühl J, Althaus K, Fürll B, Selleng S (2010). Heparin-induced thrombocytopenia: Towards standardization of platelet factor 4/heparin antigen tests. J Thromb Haemost.

[4] Martel N, Lee J, Wells PS (2005). Risk for heparin-induced thrombocytopenia with unfractionated and low-molecular-weight heparin thromboprophylaxis: a meta-analysis. Blood.

[5] Linkins L-A, Dans AL, Moores LK, Bona R, Davidson BL, Schulman S, Crowther M (2012). Treatment and prevention of heparin-induced thrombocytopenia: antithrombotic therapy and prevention of thrombosis: American College of Chest Physicians evidence-based clinical practice guidelines. Chest J.

[6] Lewis BE, Wallis DE, Berkowitz SD, Matthai WH, Fareed J, Walenga JM (2001). Argatroban anticoagulant therapy in patients with heparin-induced thrombocytopenia. Circulation.

[7] Zangrillo A, Landoni G, Biondi-Zoccai G, Greco M, Greco T, Frati G (2013). A meta-analysis of complications and mortality of extracorporeal membrane oxygenation. Crit Care Resusc.

[8] Karagiannidis C, Brodie D, Strassmann S, Stoelben E, Philipp A, Bein T (2016). Extracorporeal membrane oxygenation: evolving epidemiology and mortality. Intensive Care Med.

[9] Barbaro RP, Odetola FO, Kidwell KM, Paden ML, Bartlett RH, Davis MM, Annich GM (2015). Association of hospital-level volume of extracorporeal membrane oxygenation cases and mortality. Analysis of the extracorporeal life support organization registry. Am J Respir Crit Care Med.

[10] Ranieri VM, Rubenfeld GD, Thompson BT, Ferguson ND, Caldwell E, Fan E (2012). Acute respiratory distress syndrome: the Berlin definition. JAMA.

[11] Deja M, Hommel M, Weber-Carstens S, Moss M, von Dossow V, Sander M, Pille C, Spies C (2008). Evidence-based therapy of severe acute respiratory distress syndrome: an algorithm-guided approach. J Int Med Res.

[12] Murray JF, Matthay MA, Luce JM, Flick MR (1988). An expanded definition of the adult respiratory distress syndrome. Am Rev Resp Dis.

[13] Kim SC, Tran N, Schewe JC, Boehm O, Wittmann M, Graeff I (2015). Safety and economic considerations of argatroban use in critically ill patients: a retrospective analysis. J Cardiothorac Surg.

[14] Brun-Buisson C, Minelli C, Bertolini G, Brazzi L, Pimentel J, Lewandowski K (2004). Epidemiology and outcome of acute lung injury in european intensive care units. Results from the ALIVE study. Intensive Care Med.

[15] Balzer F, Menk M, Ziegler J, Pille C, Wernecke KD, Spies C (2016). Predictors of survival in critically ill patients with acute respiratory distress syndrome (ARDS): an observational study. BMC Anesthesiol.

[16] Vo QA, Lin JK, Tong LM (2015). Efficacy and safety of argatroban and bivalirudin in patients with suspected heparin-induced thrombocytopenia. Ann Pharmacother.

[17] Thiele T, Selleng K, Selleng S, Greinacher A, Bakchoul T (2013). Thrombocytopenia in the intensive care unit-diagnostic approach and management. Semin Hematol.

[18] Begelman SM, Baghdasarian SB, Singh IM, Militello MA, Hursting MJ, Bartholomew JR (2008). Argatroban anticoagulation in intensive care patients: effects of heart failure and multiple organ system failure. J Intensive Care Med.

[19] Beiderlinden M, Treschan T, Görlinger K, Peters J (2007). Argatroban in extracorporeal membrane oxygenation. Artif Organs.

[20] Yoon JH, Yeh RW, Nam KH, Hoffman WD, Agnihotri AK, Jang IK (2010). Safety and efficacy of the argatroban therapy during the early post-cardiac surgery period. J Thromb Thrombolysis.

[21] Saugel B, Phillip V, Moessmer G, Schmid RM, Huber W (2010). Argatroban therapy for heparin-induced thrombocytopenia in ICU patients with multiple organ dysfunction syndrome: a retrospective study. Crit Care.

[22] Thiagarajan RR, Barbaro RP, Rycus PT, Mcmullan DM, Conrad SA, Fortenberry JD (2017). Extracorporeal life support organization registry international report 2016. ASAIO J.

[23] Sklar MC, Sym E, Lequier L, Fan E, Kanji HD (2016). Anticoagulation practices during venovenous extracorporeal membrane oxygenation for respiratory failure. A systematic review. Ann Am Thorac Soc.

[24] Weingart C, Lubnow M, Philipp A, Bein T, Camboni D, Müller T (2015). Comparison of coagulation parameters, anticoagulation, and need for transfusion in patients on interventional lung assist or veno-venous extracorporeal membrane oxygenation. Artif Organs.

[25] Trudzinski FC, Minko P, Rapp D, Fähndrich S, Haake H, Haab M (2016). Runtime and aPTT predict venous thrombosis and thromboembolism in patients on extracorporeal membrane oxygenation: a retrospective analysis. Ann Intensive Care.

[26] Richard C, Argaud L, Blet A, Boulain T, Contentin L, Dechartres A (2014). Extracorporeal life support for patients with acute respiratory distress syndrome: report of a consensus conference. Ann Intensive Care.

[27] Doepker B, Mount KL, Ryder LJ, Gerlach AT, Murphy CV, Philips GS (2012). Bleeding risk factors associated with argatroban therapy in the critically ill. J Thromb Thrombolysis.

[28] Aubron C, Cheng AC, Pilcher D, Leong T, Magrin G, Cooper DJ (2013). Factors associated with outcomes of patients on extracorporeal membrane oxygenation support: a 5-year cohort study. Crit Care.

[29] Treichl B, Bachler M, Lorenz I, Friesenecker B, Oswald E, Schlimp CJ (2015). Efficacy of argatroban in critically ill patients with heparin resistance: a retrospective analysis. Semin Thromb Hemost.

[30] Warkentin TE (2014). Anticoagulation failure in coagulopathic patients: PTT confounding and other pitfalls. Expert Opin Drug Saf.

